# Exploring Social Support Strategies and Socio-Cultural Factors Influencing Social Isolation and Loneliness: The Role of Digital Literacy

**DOI:** 10.3390/healthcare12212149

**Published:** 2024-10-29

**Authors:** Ravi Batra, Jason D. Flatt, Jennifer R. Pharr, Manoj Sharma, Jagdish Khubchandani, Amar Kanekar, Francesco Chirico, Kavita Batra

**Affiliations:** 1 Department of Environmental and Occupational Health, School of Public Health, University of Nevada, Las Vegas, NV 89119, USA; ravi.batra123@gmail.com (R.B.); jennifer.pharr@unlv.edu (J.R.P.); 2 Department of Information Technology and Testing Center of Excellence, Coforge, Atlanta, GA 30338, USA; 3 Department of Social and Behavioral Health, School of Public Health, University of Nevada, Las Vegas, NV 89119, USA; jason.flatt@unlv.edu (J.D.F.); manoj.sharma@unlv.edu (M.S.); 4 Department of Internal Medicine, Kirk Kerkorian School of Medicine at UNLV, University of Nevada, Las Vegas, NV 89106, USA; 5 College of Health, Education, and Social Transformation, New Mexico State University, Las Cruces, NM 88003, USA; jagdish@nmsu.edu; 6 School of Counseling, Human Performance and Rehabilitation, University of Arkansas at Little Rock, Little Rock, AR 72204, USA; axkanekar@ualr.edu; 7 Post-Graduate School of Occupational Health, Università Cattolica del Sacro Cuore, 00168 Rome, Italy; medlavchirico@gmail.com; 8 Health Service Department, Italian State Police, Ministry of the Interior, 00185 Milan, Italy; 9 Department of Medical Education, Kirk Kerkorian School of Medicine at UNLV, University of Nevada, Las Vegas, NV 89106, USA; 10 Office of Research, Kirk Kerkorian School of Medicine at UNLV, University of Nevada, Las Vegas, NV 89106, USA

**Keywords:** social isolation, loneliness, aging, COVID-19, mental health

## Abstract

Background/Objectives: Social isolation (SI) and loneliness (L) are the long-standing critical concerns impacting the mental well-being of older adults. The COVID-19 pandemic has exacerbated existing vulnerabilities, leading to a notable rise in perceived social isolation (PSI) and its associated risks among an aging population. Reportedly, nearly 35% of older Americans felt lonely before the pandemic, with the pandemic further intensifying these feelings. This commentary examines the multifaceted factors contributing to PSI, including demographic and socio-economic characteristics. Methods: We outline the health risks associated with PSI, including cardiovascular diseases and mental health conditions. Results: This commentary addresses the potential of information and communication technology (ICT) to alleviate loneliness, despite significant barriers such as the digital divide and technological anxiety among older adults. Conclusions: This commentary advocates targeted digital literacy interventions and theoretical frameworks to enhance technology adoption and mitigate PSI, ultimately aiming to improve health outcomes and quality of life for the aging population.

## 1. Social Isolation and Loneliness

Social isolation (SI) refers to the objective lack of social connectivity or a low frequency of social contact, whereas loneliness (L) is the subjective experience of being alone or feeling disconnected from others [1,2,3]. Some authors defined loneliness as an emotional state resulting from a perception that the quality and quantity of one’s social support are unable to meet an individual’s social needs [1,2,3]. In other words, loneliness can be viewed as “perceived social isolation” (hereafter PSI) rather than objective social isolation, as described by Hawkley and Cacioppo in 2010. Previous research recognized a clear distinction between these closely related concepts since 1970 [1,4]. It is possible that people with multiple social connections feel lonely; on the other hand, people who are not socially connected may not feel lonely [1,2,5]. Over a lifetime, PSI can be episodic or continuous (chronic) depending on an individual’s conditions and perceptions [6]. Commonly used tools for measuring PSI include the UCLA Loneliness Scale, which assesses feelings of loneliness and lack of companionship [7], and the Social Isolation Scale, which evaluates social engagement and perceived support [3].

On a related note, PSI and digital literacy (DL) are distinct yet interconnected concepts that can significantly impact individuals’ well-being. DL encompasses the skills required to effectively navigate and engage with digital technologies, such as evaluating information and communicating online [8]. High levels of digital literacy empower individuals to form connections and access resources, potentially mitigating feelings of isolation [9]. Tools like the Digital Literacy Assessment (DLA) developed by the European Commission assess competencies in digital skills, while the Web Literacy Test measures users’ abilities to navigate and critically engage with online content [10]. These measurement tools provide comprehensive frameworks for assessing PSI and DL, enabling researchers to explore the relationship between these constructs. By utilizing validated instruments like the UCLA Loneliness Scale and the Digital Literacy Assessment, studies can obtain reliable data to inform interventions aimed at reducing perceived social isolation through improved digital literacy.

PSI is a commonly occurring experience that gradually diminishes through middle age and then increases with age [1,3]. This phenomenon is particularly concerning among older adults globally, who are vulnerable to various health and social risk factors, including living alone, chronic illnesses, the loss of loved ones, and impaired sensory functions associated with frailty [1,6]. These risk factors have undoubtedly increased during the COVID-19 pandemic, albeit PSI was already recognized as a behavioral epidemic in the pre-COVID era [11,12]. For instance, it was reported that nearly 45% of Americans aged 60 years and older felt loneliness, and about 1/3rd of this sample reported having frequent symptoms related to loneliness in the pre-COVID era [13]. According to a survey conducted by the American Association of Retired Persons [AARP] foundation, nearly 35% of adults aged 45 years and older in the U.S. experienced distressed feelings of loneliness in the pre-pandemic period [14,15]. A study conducted in the United Kingdom found that nearly 40% of older adults reported feelings of loneliness, with this prevalence increasing among those living alone or in care homes [16]. Similarly, in Australia, a national survey revealed that nearly 11% of older individuals frequently experienced feelings of loneliness in 2022, highlighting the pervasive nature of this issue [17,18]. In Japan, the phenomenon known as “hikikomori,” which involves extreme social withdrawal, has gained attention, affecting hundreds of thousands of individuals and raising concerns about the mental health of the elderly [19]. Compared to the United States, where approximately 45% of older adults reported experiencing loneliness pre-pandemic [13], these findings underscore the global burden of PSI, indicating that loneliness is a widespread issue that transcends national boundaries. However, while the rates of reported loneliness are significant in both contexts, cultural factors and social support systems may shape the experience and consequences of isolation differently across countries. The social distancing mandates instituted during the COVID-19 pandemic exacerbated the PSI burden among older adults, who are known to have pre-existing social vulnerabilities as opposed to their younger counterparts [1,6,12,20,21]. Reportedly, in 2020, nearly one-quarter of adults over the age of 65 years in the United States (U.S.) experienced social isolation, and about 4% experienced severe social isolation [1,21]. These data underscore the significant mental health challenges faced by older adults during the COVID-19 pandemic. It is particularly compelling to consider how the severity of these issues might have been exacerbated had the pandemic occurred before the year 2000. Prior to the widespread availability of digital communication tools, older adults encountered substantial barriers to maintaining social connections during periods of isolation. Research has shown that technology plays a crucial role in sustaining social interactions, which can help mitigate feelings of loneliness [22]. By highlighting the transformative impact of digital tools on social support, we can underscore their essential role in enhancing mental well-being during crises.

## 2. Burden and Risk Factors of PSI

Among the possible factors associated with PSI, the size and diversity of the social network along with demographic and mental health variables were noted as important predictors [15]. Several factors, including gender, sexual orientation, gender identity, marital status (being unmarried), lower education, and socio-economic status were cited as important correlates of PSI by previous reports [15]. Certain demographic groups, e.g., LGBTQ+, experience more loneliness compared to their heterosexual counterparts due to stigma, discrimination, and lack of access to healthcare [6,23]. Other groups that are often overlooked include immigrants, especially first-generation immigrants, who experience loneliness due to language barriers, differences at the community level, and family dynamics [6,23]. Some studies reported that the association between psychiatric morbidities and PSI can be bidirectional, which means that PSI can follow or precede depression and anxiety, thus indicating a reciprocal relationship [24,25,26,27,28]. Moreover, several longitudinal and cross-sectional studies indicated that the reciprocal relationships between psychiatric conditions and PSI compound over time, which further explains an increased burden of PSI among older adults [24,25,26,27,28]. Factors at several levels, including individual, psychological, social, environmental, and those associated with life course variations have been reported to have a strong association with PSI [24,25,26,27,28,29,30]. Among the social factors, meaningful social contacts, higher frequency of contacts, neighborhood attachment, and social participation were noted to have a protective role in enhancing resilience or reducing PSI among older adults [29,30].

## 3. Pathophysiology and Health Risks of PSI

The literature is still not clear if social isolation and loneliness affect health differently or if loneliness acts as a mediator for social isolation resulting in adverse health outcomes [31]. Interactions between genes and the environment have also been studied; however, the research landscape is still evolving [31]. Meta-analytical evidence from 148 longitudinal studies revealed that PSI combined offers a multifaceted measure of social connection, which builds a strong framework rather than the unidimensional measures [1,32]. PSI increases the odds of premature mortality by 50% among individuals with weaker social connections. In collectivist cultures, particularly in developing countries, social networks often act as essential support systems, potentially mitigating the negative impacts of isolation [33]. Additionally, PSI increases the risk of cardiovascular diseases by nearly 1/3rd, and this association is independent of the cardiovascular risk factors [1,32,34,35,36]. PSI is a risk factor for myocardial infection and is independently associated with poor prognosis and mortality among patients with cardiac disorders. The impact further translates to the increased burden of hospital readmissions, thereby increasing healthcare utilization and associated costs [32,34,35,36]. Collective evidence suggests that PSI has a significant impact on mental health and quality of life, which shows pronounced impacts among older adults [1]. These effects are mediated through behavioral, psychological, and biological mechanisms [1]. The molecular or biological pathways of PSI impacts on health are not well studied; however, based on some studies using animal models, it was found that increased levels of PSI lead to higher levels of inflammatory biomarkers, including C-reactive protein, fibrinogens, and interleukin-6, which increase the risk for cardiovascular diseases [31,37,38]. Pertaining to the psychological mechanism, higher levels of PSI increase the physiological stress response, which is in turn associated with higher odds of developing anxiety and depression [1]. Lastly, the quality and quantity of the social network are associated with behavioral or lifestyle factors, for instance, higher levels of PSI were associated with insomnia, physical inactivity, and daily smoking [1,39]. These concerns are more pronounced in the aging population, especially in a world where social interactions are becoming increasingly virtual due to the use of technology; older adults are known to have limited digital literacy and technology access [40].

## 4. Technology Use and Older Adults

Research suggests that information and communication technology (ICT) can help overcome PSI and associated challenges [40]. In 2016, Chopik described the role of technology in promoting physical health (with fewer chronic conditions), interpersonal relationships, psychosocial health, and subjective well-being among older adults [41]. Moreover, the use of technology can alleviate feelings of loneliness, as suggested by a multitude of peer-reviewed studies [42]. From a practical standpoint, social networking through technology has proven effective in promoting good behaviors, including acceptability or adherence to new treatments, which could offer fast recovery following an illness [43]. Despite the known benefits of technology use, there were significant disparities in technology and internet use among younger generations and older adults (“digital divide”) [41]. Previous research indicated that older adults are less receptive to new technology and feel anxious about security concerns [44,45]. Barriers were subclassified as age-related, individual or personal, and related to electronic appliances or limited access to technology. In addition, because of safety concerns and unfavorable attitudes toward technology use, some older adults believe that electronic devices release radiation, which may be detrimental to health [46,47,48]. Table 1 describes barriers to technology use among older adults [47,48,49,50,51].

Among the barriers listed above, a few of them are modifiable and can be intervened with through appropriate digital literacy and educational interventions. One study by Vaportzis et al. in 2017 found that older adults are eager to learn or adopt new technology; however, the adoption of technology was mediated by other factors, including cognitive abilities, computer anxiety, and confidence in using electronic devices [51,52]. According to data reported by the American Association of Retired Persons (AARP) in 2002, older adults over the age of 65 reported a lack of confidence in using computers [50]. There are several conceptual frameworks cited in the literature, which were utilized to improve digital literacy among older adults [53,54]. The most commonly used model was the self-efficacy model, which seemed to play a critical role in improving digital literacy levels among older adults [53]. Another barrier is “Digital Redlining,” which results from unequal access to digital tools [55].

## 5. Current State of Digital Literacy Interventions and Their Conceptual Frameworks

Digital literacy is defined as the ability to effectively find, evaluate, use, and communicate information in various digital formats. This includes proficiency in using digital devices, understanding online safety and privacy, and critically assessing the credibility of online sources [56]. Digital interventions are structured programs that utilize digital technologies to deliver therapeutic or preventive strategies aimed at improving health outcomes. These interventions can include mobile health applications, online therapy sessions, and web-based educational resources. Planning a digital intervention involves defining the target population, identifying specific health outcomes, selecting appropriate technology platforms, and ensuring user engagement throughout the process [57,58]. Some countries have specific programs and successful models of digital literacy interventions to address PSI. For instance, in the United Kingdom, the Silver Line initiative offers a free, 24/7 helpline for older adults, addressing loneliness and connecting them with services that enhance their digital skills [59]. Similarly, the TechBoomers program in the U.S. provides online courses tailored for seniors, focusing on digital literacy and helping them navigate various technologies [60]. In Australia, the Be Connected program aims to increase the digital skills of older Australians, promoting social engagement through technology [61]. These programs illustrate how targeted digital literacy interventions can mitigate social isolation and improve mental well-being among older adults.

Several theoretical models, including the Health Belief Model (HBM), the Self-efficacy Model, a combined model consisting of the Technology Acceptance Model (TAM), and the Diffusions of Innovations (DOI) models were used as the basis of digital literacy interventions [62,63,64,65,66]. Some studies also used the Social Interdependence Theory, Cognitive Theory of Multimedia, and Knowles’ theory [53,67]. Among all these frameworks, the Self-efficacy Model was the most commonly used [53]. Shi et al. (2021) examined factors at all levels, which could serve as a basis for developing a multilevel intervention based on the socio-ecological model explained in Table 2 [54,68]. This recognizes the importance of developing a multifaceted program or digital literacy intervention [54,68,69]. In 2022, McCall et al. [55] proposed a socio-ecological approach to address digital redlining in the U.S. (Table 2). The socio-ecological model highlights various levels influencing digital literacy, beginning with the individual or intrapersonal level. Factors such as age, gender, education level, socioeconomic status, and urbanization play crucial roles in shaping one’s ability to engage with technology [55]. Socioeconomic status often influences both access to technology and the ability to engage with digital literacy programs. Tailoring interventions to address these disparities can lead to better health outcomes and reduce feelings of social isolation [70]. In rural areas, access to technology and internet connectivity can be significant barriers, highlighting the need for targeted initiatives that provide resources and training specifically designed for these populations [27]. Additionally, physical and mental health status, frequency of internet use, and perceptions of online sources further impact an individual’s digital skills and confidence [64,70,71]. At the interpersonal level, relationships significantly influence digital literacy. Marital status, family dynamics, and friendships can either encourage or hinder technological adoption. Support from partners, family members, and friends can provide the motivation and resources needed to improve digital skills. Social factors also contribute to digital literacy, with social networks and community support structures facilitating learning and engagement. Generational differences in comfort with technology can create disparities in digital skills, while the overall community environment can offer essential resources and training programs [71]. Research also shows that culturally relevant programs can significantly enhance engagement and outcomes among older adults from diverse backgrounds [72]. These programs can be designed to respect and incorporate the unique cultural values, beliefs, and practices of various communities, thereby fostering a sense of belonging and relevance. Culturally relevant interventions can include tailored communication strategies, the incorporation of familiar cultural symbols and narratives, and the provision of services in the preferred language of the participants. For instance, programs that celebrate cultural traditions or include community leaders can create a more welcoming environment, encouraging older adults to participate actively [73]. Additionally, culturally relevant programs often address specific barriers that different groups face, such as mistrust in healthcare systems, socioeconomic challenges, and varying levels of access to technology. By acknowledging these factors, such programs can improve outreach and engagement, ultimately leading to better health outcomes and reduced feelings of isolation [74]. Furthermore, when older adults see their identities and experiences reflected in the programs offered to them, they are more likely to engage with the material and the facilitators, which can lead to increased social interaction and support. This engagement not only enhances individual well-being but also strengthens community ties, creating a supportive network that is vital for mental health [75].

Cultural attitudes are another important factor. Acceptance of technology, communication styles, and values surrounding community and relationships vary across cultures, affecting how individuals interact with digital tools. Language barriers and religious beliefs can further influence engagement and access to technology. Organizational factors play a vital role as well. A supportive organizational culture, strong leadership, and adequate resource availability can promote digital literacy initiatives and training opportunities. Finally, policy and environmental factors such as community infrastructure, equitable access to technology, and education programs create the necessary framework for enhancing digital skills among diverse populations.

These comprehensive influences underscore the need for multi-level approaches to improve digital literacy, as described in Table 2.

## 6. Conclusions

Social isolation and loneliness are long-standing concerns among older adults, and the COVID-19 pandemic has undoubtedly exaggerated these concerns [76]. Previous research suggests that the likelihood of technology adoption decreases with age, and efficient training environments can increase the self-efficacy to use computers among older adults, which will promote their quality of life and yields better mental health outcomes [77]. Through this review, the authors would like to advocate for developing appropriate multilevel and culturally appropriate digital literacy interventions to address the “digital divide” among older adults in the U.S. [78]. Policymakers must prioritize support systems that target not only digital literacy but also broader social and economic inequalities. Older adults without adequate social and financial support are particularly vulnerable, and targeted healthcare policies should aim to reduce these disparities. Providing accessible community-based programs, improving affordable healthcare, and promoting initiatives that foster social inclusion and resilience are critical steps toward mitigating the long-term impacts of PSI. Such policies would not only enhance the quality of life for older adults but also reduce the broader societal burden of social isolation and loneliness [79,80,81,82]. Additionally, psychological interventions, including home-based therapies like animal-assisted therapy, have shown significant promise in combating the emotional effects of isolation, particularly during the COVID-19 pandemic [75]. These interventions provide companionship and emotional support, reducing anxiety and depressive symptoms [83]. Implementing such programs as part of home care services for older adults could serve as a valuable tool in reducing the psychological and emotional burdens associated with prolonged isolation [83]. Expanding access to these services, especially for those with limited social connections, is essential for a holistic approach to addressing PSI.

## Figures and Tables

**Table 1:** Barriers to technology use among older adults.

Type of Barrier	Examples
Age-related	Physical, mental, and cognitive limitations
Individual/personal barriers	Lack of confidence, educational limitations (low digital literacy), or lack of knowledge
Barriers to learning	Lack of motivation, time, and resources for learning
Limited access to technology	Cost of purchasing the device and its maintenance
Environmental or living conditions	e.g., older adults living in rental homes or small homes
Unfavorable attitudes	Harmful radiation from devices, reduced interpersonal interactions, suitability of technology with younger people, privacy concerns
Appliance-related barriers	Structural limitations: Product appearance (small buttons, inappropriate font size, and color), touchscreen Instructional limitations: Difficult set up, confusing instructions, need assistance with use, lack of detailed or clear instructions

Adapted from [47,48,51].

**Table 2:** Factors to consider when developing digital literacy intervention based on the socio-ecological model proposed by McCall et al. in 2022 [55].

Level of the Socio-Ecological Model	Factors That Will Influence Digital Literacy
Individual or intrapersonal level	Age, gender, education level, socioeconomic status, urbanization level, physical and mental health status, frequency of internet use, perception towards online sources
Interpersonal level	Marital status, family members, friends
Social level	Social networks, generational differences, community support structure
Cultural level	Cultural attitudes toward technology, communication style, value of community and relationships, religious and spiritual beliefs, language barriers
Organizational level	Organizational culture, leadership support, resource availability
Policy/Environmental level	Community infrastructure, equitable access to technology, workplace policies, education and training programs, cultural competency in policies

## Data Availability

Data sharing is not applicable.

## References

[1] Donovan N.J., Blazer D.G. (2020). Social isolation and loneliness in older adults: Review and commentary of a National Academies report. Am. J. Geriatr. Psychiatry.

[2] Emerson E., Fortune N., Llewellyn G., Stancliffe R. (2021). Loneliness, social support, social isolation and wellbeing among working age adults with and without disability: Cross-sectional study. Disabil. Health J..

[3] Hawkley L.C., Cacioppo J.T. (2010). Loneliness matters: A theoretical and empirical review of consequences and mechanisms. Ann. Behav. Med..

[4] Blazer D.G. (1982). Social support and mortality in an elderly community population. Am. J. Epidemiol..

[5] Cacioppo J.T., Cacioppo S. (2014). Older adults reporting social isolation or loneliness show poorer cognitive function 4 years later. Evid. Based Nurs..

[6] National Academies of Sciences, Engineering, Medicine, Division of Behavioral and Social Sciences and Education, Health and Medicine Division, Board on Behavioral, Cognitive, Sensory Sciences, Board on Health Sciences Policy, Committee on the Health and Medical Dimensions of Social Isolation and Loneliness in Older Adults (2020). Social Isolation and Loneliness in Older Adults: Opportunities for the Health care System.

[7] Russell D.W. (1996). UCLA Loneliness Scale (Version 3): Reliability, validity, and factor structure. J. Pers. Assess..

[8] Eshet-Alkalai Y. (2020). Digital literacy: A conceptual framework for survival skills in the digital era. J. Educ. Multimed. Hypermedia.

[9] Dong Q., Liu T., Liu R., Yang H., Liu C. (2023). Effectiveness of digital health literacy interventions in older adults: Single-arm meta-analysis. J. Med. Internet Res..

[10] European Commission. Digital Competence Framework 2.0. Joint Research Centre. 2019. https://joint-research-centre.ec.europa.eu/oldpage-digcomp/digcomp-framework_en.

[11] Na P.J., Straus E., Tsai J., Norman S.B., Southwick S.M., Pietrzak R.H. (2022). Loneliness in U.S. military veterans during the COVID-19 pandemic: A nationally representative, prospective cohort study. J. Psychiatr. Res..

[12] Hwang T.J., Rabheru K., Peisah C., Reichman W., Ikeda M. (2020). Loneliness and social isolation during the COVID-19 pandemic. Int. Psychogeriatr..

[13] Perissinotto C.M., Stijacic Cenzer I., Covinsky K.E. (2012). Loneliness in older persons: A predictor of functional decline and death. Arch. Intern. Med..

[14] American Association of Retired Persons (AARP) (2002). Staying Ahead of the Curve: The AARP Work and Career Study.

[15] Thayer C., Anderson G.O. (2018). Loneliness and Social Connections: A National Survey of Adults 45 and Older.

[16] Victor C.R., Rippon I., Barreto M., Hammond C., Qualter P. (2022). Older adults’ experiences of loneliness over the lifecourse: An exploratory study using the BBC loneliness experiment. Arch. Gerontol. Geriatr..

[17] Australian Institute of Health and Welfare (2022). Social Isolation and Loneliness. https://www.aihw.gov.au/mental-health/topic-areas/social-isolation-and-loneliness.

[18] Nicholson N.R. (2012). A review of social isolation: An important but underassessed condition in older adults. J. Prim. Prev..

[19] Teo A.R., Gaw A.C. (2010). Hikikomori, a Japanese culture-bound syndrome of social withdrawal? A proposal for DSM-5. J. Nerv. Ment. Dis..

[20] Andrew M.K., Mitnitski A., Rockwood K. (2008). Social vulnerability, frailty and mortality in elderly people. PLoS ONE.

[21] Cudjoe T., Roth D.L., Szanton S.L., Wolff J.L., Boyd C.M., Thorpe R.J. (2020). The epidemiology of social isolation: National health and aging trends study. J. Gerontol. B Psychol. Sci. Soc. Sci..

[22] Cattan M., White M., Bond J., Learmouth A. (2005). Preventing social isolation and loneliness among older people: A systematic review of health promotion interventions. Ageing Soc..

[23] Centers for Disease Control and Prevention (2021). Loneliness and Social Isolation Linked to Serious Health Conditions. https://www.cdc.gov/social-connectedness/risk-factors/index.html.

[24] Domènech-Abella J., Mundó J., Haro J.M., Rubio-Valera M. (2019). Anxiety, depression, loneliness and social network in the elderly: Longitudinal associations from the Irish Longitudinal Study on Ageing (TILDA). J. Affect. Disord..

[25] Falk Dahl C.A., Dahl A.A. (2010). Lifestyle and social network in individuals with high level of social phobia/anxiety symptoms: A community-based study. Soc. Psychiatry Psychiatr. Epidemiol..

[26] Luo Y., Hawkley L.C., Waite L.J., Cacioppo J.T. (2012). Loneliness, health, and mortality in old age: A national longitudinal study. Soc. Sci. Med..

[27] Lim M.H., Rodebaugh T.L., Zyphur M.J., Gleeson J.F. (2016). Loneliness over time: The crucial role of social anxiety. J. Abnorm. Psychol..

[28] Schwarzbach M., Luppa M., Forstmeier S., König H., Riedel-Heller S.G. (2013). Social relations and depression in late life: A systematic review. Int. J. Geriatr. Psychiatry.

[29] Kemperman A., van den Berg P., Weijs-Perrée M., Uijtdewillegen K. (2019). Loneliness of older adults: Social network and the living environment. Int. J. Environ. Res. Public Health.

[30] Park S., Kim T.H., Eom T.R. (2021). Impact of social network size and contact frequency on resilience in community-dwelling healthy older adults living alone in the Republic of Korea. Int. J. Environ. Res. Public Health.

[31] National Institute on Aging (2019). Social Isolation, Loneliness in Older People Pose Health Risks. https://www.nia.nih.gov/news/social-isolation-loneliness-older-people-pose-health-risks.

[32] Holt-Lunstad J., Smith T.B., Layton J.B. (2010). Social relationships and mortality risk: A meta-analytic review. PLoS Med..

[33] Ribeiro-Gonçalves J.A., Costa P.A., Leal I. (2023). Loneliness, ageism, and mental health: The buffering role of resilience in seniors. Int. J. Clin. Health Psychol..

[34] Hakulinen C., Pulkki-Råback L., Virtanen M., Jokela M., Kivimäki M., Elovainio M. (2018). Social isolation and loneliness as risk factors for myocardial infarction, stroke, and mortality: UK Biobank cohort study of 479,054 men and women. Heart.

[35] Manemann S., Chamberlain A.M., Roger V.L., Griffin J.M., Boyd C.M., Cudjoe T.K., Jensen D., Weston S.A., Fabbri M., Jiang R. (2018). Perceived social isolation and outcomes in patients with heart failure. J. Am. Heart Assoc..

[36] Valtorta N.K., Kanaan M., Gilbody S., Ronzi S., Hanratty B. (2016). Loneliness and social isolation as risk factors for coronary heart disease and stroke: Systematic review and meta-analysis of longitudinal observational studies. Heart.

[37] Bhatti A.B., Haq A.U. (2017). The pathophysiology of perceived social isolation: Effects on health and mortality. Cureus.

[38] Cole S.W., Capitanio J.P., Chun K., Arevalo J.M., Ma J., Cacioppo J.T. (2015). Myeloid differentiation architecture of leukocyte transcriptome dynamics in perceived social isolation. Proc. Natl. Acad. Sci. USA.

[39] Christiansen J., Larsen F.B., Lasgaard M. (2016). Do stress, health behavior, and sleep mediate the association between loneliness and adverse health conditions among older people?. Soc. Sci. Med..

[40] Latikka R., Rubio-Hernández R., Lohan E.S., Rantala J., Nieto Fernández F., Laitinen A., Oksanen A. (2021). Older adults’ loneliness, social isolation, and physical information and communication technology in the era of ambient assisted living: A systematic literature review. J. Med. Internet Res..

[41] Chopik W.J. (2016). The benefits of social technology use among older adults are mediated by reduced loneliness. Cyberpsychol. Behav. Soc. Netw..

[42] Heo J., Chun S., Lee S., Lee K.H., Kim J. (2015). Internet use and well-being in older adults. Cyberpsychol. Behav. Soc. Netw..

[43] Smith J.A., Johnson L.R., Patel R., Thompson K.M. (2022). Family influence on chronic pain management: The role of encouragement and support in patient adherence. J. Health Psychol..

[44] Laguna K., Babcock R.L. (1997). Computer anxiety in young and older adults: Implications for human-computer interactions in older populations. Comput. Hum. Behav..

[45] Vroman K.G., Arthanat S., Lysack C. (2015). “Who over 65 is online?” Older adults’ dispositions toward information communication technology. Comput. Hum. Behav..

[46] Busch P.A., Hausvik G.I., Ropstad O.K., Pettersen D. (2021). Smartphone usage among older adults. Comput. Hum. Behav..

[47] Harris M.T., Blocker K.A., Rogers W.A. (2022). Older adults and smart technology: Facilitators and barriers to use. Front. Comput. Sci..

[48] Yazdani-Darki M., Rahemi Z., Adib-Hajbaghery M., Izadi F.S. (2020). Older adults’ barriers to using technology in daily life: A qualitative study. Nurs. Midwifery Stud..

[49] Mitzner T.L., Boron J.B., Fausset C.B., Adams A.E., Charness N., Czaja S.J., Dijkstra K., Fisk A.D., Rogers W.A., Sharit J. (2010). Older adults talk technology: Technology usage and attitudes. Comput. Hum. Behav..

[50] Mitzner T.L., Savla J., Boot W.R., Sharit J., Charness N., Czaja S.J., Rogers W.A. (2019). Technology adoption by older adults: Findings from the PRISM trial. Gerontologist.

[51] Vaportzis E., Clausen M.G., Gow A.J. (2017). Older adults’ perceptions of technology and barriers to interacting with tablet computers: A focus group study. Front. Psychol..

[52] Czaja S.J., Charness N., Fisk A.D., Hertzog C., Nair S.N., Rogers W.A., Sharit J. (2006). Factors predicting the use of technology: Findings from the Center for Research and Education on Aging and Technology Enhancement (CREATE). Psychol. Aging.

[53] Pourrazavi S., Kouzekanani K., Bazargan-Hejazi S., Shaghaghi A., Hashemiparast M., Fathifar Z., Allahverdipour H. (2020). Theory-based e-health literacy interventions in older adults: A systematic review. Arch. Public Health..

[54] Shi Y., Ma D., Zhang J., Chen B. (2021). In the digital age: A systematic literature review of e-health literacy and influencing factors among Chinese older adults. Z. Gesundh. Wiss..

[55] McCall T., Asuzu K., Oladele C.R., Leung T.I., Wang K.H. (2022). A socio-ecological approach to addressing digital redlining in the United States: A call to action for health equity. Front. Digit. Health.

[56] Arias López M.D., Ong B.A., Borrat Frigola X., Fernández A.L., Hicklent R.S., Obeles A.J., Rocimo A.M., Celi L.A. (2023). Digital literacy as a new determinant of health: A scoping review. PLoS Digit. Health.

[57] Welch V., Mathew C.M., Babelmorad P., Li Y., Ghogomu E.T., Borg J., Conde M., Kristjansson E., Lyddiatt A., Marcus S. (2021). Health, social care, and technological interventions to improve functional ability of older adults living at home: An evidence and gap map. Campbell. Syst. Rev..

[58] Welch V., Ghogomu E.T., Barbeau V.I., Dowling S., Doyle R., Beveridge E., Boulton E., Desai P., Huang J., Elmestekawy N. (2023). Digital interventions to reduce social isolation and loneliness in older adults: An evidence and gap map. Campbell. Syst. Rev..

[59] The Silver Line About Us. https://www.thesilverline.org.uk/#:~:text=What%20is%20The%20Silver%20Line,feelings%20of%20loneliness%20and%20isolation.

[60] TechBoomers About Us. https://techboomers.com/about.

[61] Be Connected About Us. https://beconnected.esafety.gov.au/.

[62] Chang S.J., Jang S.J., Lee H., Kim H. (2021). Building on evidence to improve eHealth literacy in older adults: A systematic review. Comput. Inform. Nurs..

[63] Chen K., Chan A.H.S. (2014). Gerontechnology acceptance by elderly Hong Kong Chinese: A senior technology acceptance model (STAM). Ergonomics.

[64] Chiu C., Hu Y., Lin D., Chang F.Y., Chang C.S., Lai C.F. (2016). The attitudes, impact, and learning needs of older adults using apps on touchscreen mobile devices: Results from a pilot study. Comput. Hum. Behav..

[65] Chu A., Huber J., Mastel-Smith B., Cesario S. (2009). Partnering with seniors for better health: Computer use and Internet health information retrieval among older adults in a low socioeconomic community. J. Med. Libr. Assoc..

[66] Fink A., Beck J.C. (2015). Developing and evaluating a website to guide older adults in their health information searches: A mixed-methods approach. J. Appl. Gerontol..

[67] Xie B. (2011). Effects of an eHealth literacy intervention for older adults. J. Med. Internet Res..

[68] Sharma M. (2022). Theoretical Foundations of Health Education and Health Promotion.

[69] Koehle H., Kronk C., Lee Y.J. (2022). Digital health equity: Addressing power, usability, and trust to strengthen health systems. Yearb. Med. Inform..

[70] Yoon H., Jang Y., Vaughan P.W., Garcia M. (2020). Older adults’ internet use for health information: Digital divide by race/ethnicity and socioeconomic status. J. Appl. Gerontol..

[71] Jaworski B.K., Webb Hooper M., Aklin W.M., Jean-Francois B., Elwood W.N., Belis D., Riley W.T., Hunter C.M. (2023). Advancing digital health equity: Directions for behavioral and social science research. Transl. Behav. Med..

[72] Tierney S., Libert S., Gorenberg J., Wong G., Turk A., Husk K., Chatterjee H.J., Eccles K., Potter C., Webster E. (2022). Tailoring cultural offers to meet the needs of older people during uncertain times: A rapid realist review. BMC Med..

[73] Chowdhury D., Baiocco-Romano L., Sacco V., El Hajj K., Stolee P. (2022). Cultural competence interventions for health care providers working with racialized foreign-born older adults: Protocol for a systematic review. JMIR Res. Protoc..

[74] Brach C., Fraser I. (2002). Reducing disparities through culturally competent health care: An analysis of the business case. Qual. Manag. Health Care.

[75] Finistrella M., Flores P., Frediani G. (2024). Animal-assisted therapy in patients affected by schizophrenia and schizophrenic-related disorders: A scoping review. Adv. Med. Psychol. Public Health.

[76] Hood S., Campbell B., Baker K. (2023). Culturally Informed Community Engagement: Implications for Inclusive Science and Health Equity.

[77] Van Deursen A.J., Helsper E.J. (2015). The role of digital skills in internet use and social participation among older adults. Ageing Soc..

[78] Giampà A. (2023). Embodied online therapy: The efficacy of somatic and psychological treatment delivered digitally. Adv. Med. Psychol. Public Health.

[79] Gharib M., Borhaninejad V., Rashedi V. (2024). Mental health challenges among older adults. Adv. Med. Psychol. Public Health.

[80] Poudel P. (2024). Prevalence and determinants of income among people with disabilities in Nepal: A cross-sectional study. Adv. Med. Psychol. Public Health.

[81] Zhussipbek G. (2024). The human right to health: Empathy and universal human dignity versus apathetic capitalism. Adv. Med. Psychol. Public Health.

[82] Lim H.A., Lee J.S.W., Lim M.H., Teo L.P.Z., Sin N.S.W., Lim R.W., Chua S.M., Yeo J.Q., Ngiam N.H.W., Tey A.J.Y. (2022). Bridging connectivity issues in digital access and literacy: Reflections on empowering vulnerable older adults in Singapore. JMIR Aging.

[83] McHugh Power J., Hannigan C., Hyland P., Brennan S., Kee F., Lawlor B.A. (2020). Depressive symptoms predict increased social and emotional loneliness in older adults. Aging Ment. Health.

